# A global temperature control of silicate weathering intensity

**DOI:** 10.1038/s41467-022-29415-0

**Published:** 2022-04-04

**Authors:** Kai Deng, Shouye Yang, Yulong Guo

**Affiliations:** 1 State Key Laboratory of Marine Geology, Tongji University, 200092 Shanghai, China; 2 Institute of Geochemistry and Petrology, Department of Earth Sciences, ETH Zürich, Clausiusstrasse 25, 8092 Zürich, Switzerland

**Keywords:** Carbon cycle, Carbon cycle

## Abstract

Silicate weathering as an important negative feedback can regulate the Earth’s climate over time, but much debate concerns its response strength to each climatic factor and its evolution with land surface reorganisation. Such discrepancy arises from lacking weathering proxy validation and scarce quantitative paleo-constraints on individual forcing factors. Here we examine the catchment-scale link of silicate weathering intensity with various environmental parameters using a global compilation of modern sediment dataset (*n* = 3828). We show the primary control of temperature on silicate weathering given the monotonic increase of feldspar dissolution with it (0–30 °C), while controls of precipitation or topographic-lithological factors are regional and subordinate. We interpret the non-linear forcing of temperature on feldspar dissolution as depletion of more reactive plagioclase (relative to orthoclase) at higher temperature. Our results hint at stronger temperature-weathering feedback at lower surface temperature and support the hypothesis of increased land surface reactivity during the late Cenozoic cooling.

## Abstract (web summary)

How silicate weathering responds to and regulates Earth’s climate remain controversial. This study suggests the primary control of temperature on weathering intensity globally and the temperature-weathering feedback may be stronger in cold Earth.

## Introduction

Chemical weathering of silicate minerals plays a major role in maintaining the long-term habitability of Earth’s climate over geological timescales via a negative feedback mechanism^1–4^. One long-standing hypothesis on such feedback is that an increase in volcanic CO_2_ emission causes a rise of surface temperature through the greenhouse effect; meanwhile, a higher temperature can enhance silicate weathering that, in turn, leads to the draw-down of atmospheric CO_2_^5,6^. As such, the climate-weathering feedback hypothesis depends critically on the response strength of silicate weathering to temperature.

Although temperature-dependence of silicate weathering has been observed in laboratory experiments^7^ and field studies on small watersheds^8–10^, it is commonly obscured over large spatial or temporal scales due to covariation of other factors with temperature or minor temperature variability. Many studies then seek other forcing mechanisms to interpret climate-weathering feedback, including hydrologic regulation (precipitation and runoff)^11^, tectonic uplift (relief and physical erosion)^2,3^, and reorganization of land surface^1,12^. However, validation of these hypotheses over geological timescales is always challenging, because (1) paleo-weathering records may not be robust given the inconsistency in trends among various weathering proxies^1,4^ and (2) high-quality paleo-records on any of these individual forcing factors (rather than mixed signal like deep-sea δ^18^O^13^) are scarce for comparison.

As the present is a key to the past, one approach to test the link between environmental forcing factors and silicate weathering is to focus on an abundant modern dataset that covers a large environmental gradient. Two weathering proxies based on siliciclastic sediments, that are chemical index of alteration (CIA)^14,15^ and weathering index of Parker (WIP)^16^ stand out as they are the most widely applied proxies due to their simple methodology and requirement of only major element measurements^15,17^. CIA is the proportion of immobile Al_2_O_3_ versus labile oxides (CaO, Na_2_O, and K_2_O) in the silicate fraction, while WIP is the sum of molar proportions of labile elements (Ca, Mg, Na, and K) weighted by their susceptibility to weathering (Methods). Both indices indicate the intensity of silicate weathering and partly track the hydrolysis reaction of feldspar^18^, which is the most abundant rock-forming mineral (53% of upper-crust)^18^. With an increase of weathering intensity, mobile cations in feldspar release into the dissolved phase, and immobile elements remain in solid residue by forming clay minerals^19^. As such, CIA and WIP increase and decrease, respectively^14,16^.

**Fig. 1:**
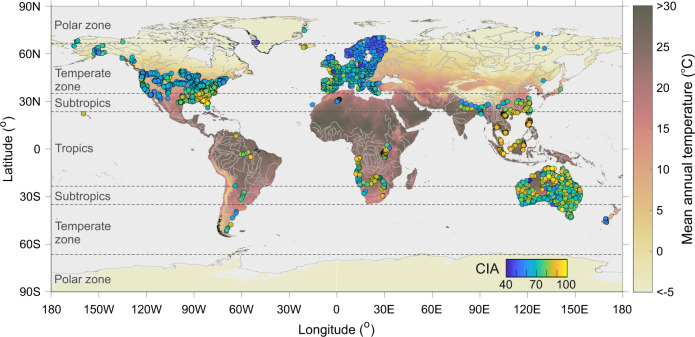
Geographic locations of compiled weathering index data in fine-grained sediments. The symbol color indicates the value of the chemical index of alteration (CIA; *n* = 3828). Data source of CIA is described in Methods. A sub-sample set (*n* = 2989) collected from small- and median-sized catchments was used for geospatial analysis and discussion on environmental controls (Methods). The global map of mean annual temperature is shown for comparison. At first glance, high CIA values (yellow symbols) are in general limited to the tropics and subtropics.

In this study, we compiled a major element dataset of fine-grained sediments from modern rivers (*n* = 3828) across six continents for CIA-WIP calculation (Fig. 1). We only discuss CIA in the main text for conciseness, as the WIP dataset provided in Supplementary Information generally derives consistent trends. Corresponding basin-scale environmental forcing factors (*n* = 20) were extracted to assess controls of silicate weathering and the potential of weathering index as a paleoclimate proxy.

## Results

### Latitudinal variations of silicate weathering index

We restricted our analysis to silt- and clay-sized sediments to minimize mineralogical differentiation by hydraulic sorting^14^. For compiled sediment dataset, we calculated CIA values based on molar proportions of silicate-bound major elements^15^ (Methods). In principle, CIA varies within a restricted range around 40–50 for fresh bedrock^20^ and increases towards 100 during extreme weathering with e.g., kaolinite as the only weathering product.

**Fig. 2:**
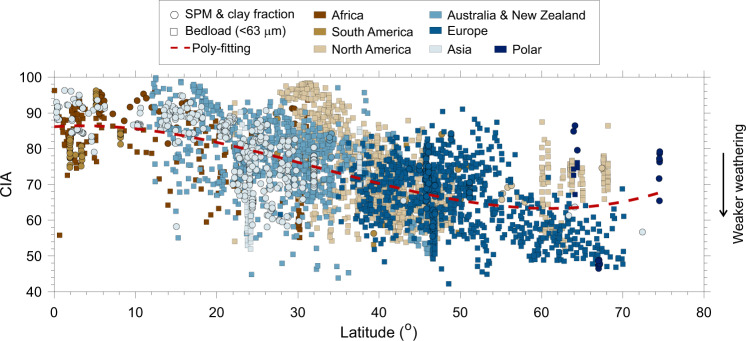
Latitudinal distribution of CIA in fine-grained sediments. Sample types include surface suspended particulate matter (SPM), clay fraction of sediments and bedload sieved to <63 μm. The latitude of sampling locations shown on the X-axis equally refers to the southern and northern hemispheres, respectively. The polynomial fitting between latitude and weathering index is shown as a red dashed line. CIA generally decreases with latitude. The latitudinal distribution of WIP is provided in Supplementary Fig. 1. “Polar” symbols here include samples from Greenland and Iceland.

We first observe a systematic decreasing trend in CIA with latitude (Fig. 2), suggesting stronger weathering towards the tropics. As expected, low-latitude (<10°) regions are characterized by high CIA (mostly 80–100), while weathering index values at latitudes of >60° are closer to those of fresh rocks (CIA as low as ~50). Such decreasing trend with latitude is consistent among continents (especially at <60°), hinting at a common controlling mechanism. At high latitudes, samples from North America and polar regions clearly show higher CIA compared to other samples. We attribute such difference (*p* < 0.01 from two-sided Wilcoxon rank-sum test) to control of diverse environmental forcings (see next sub-section).

The similarly latitudinal trend in silicate weathering intensity has been observed in soil profiles^21,22^, river sediments^17,23^, and paleo-sedimentary archives^24^, and interpreted as controls of latitude-related climate variability. We confirm such large-scale latitudinal dependence based on a dataset of the highest spatial resolution ever (*n* > 3 × 10^3^ compared to *n* < 10^2^ in some other studies). Furthermore, the inverse correlation between CIA and latitudes echoes the pioneering study of Nesbitt and Young^14^ (1982) that interpreted CIA variations in the Proterozoic as changes in latitudes of depositional locations and thus in climate. Nevertheless, our observation on the latitudinal trend of CIA does not necessarily suggest any causal link between them, and the CIA is in fact controlled by environmental forcing factors as discussed in detail below.

### Revisiting environmental controls of weathering intensity

Given the wide spatial coverage of our compiled dataset and thus the large gradient of each environmental variable (Table 1), we can robustly evaluate the major controls of silicate weathering intensity. Generally, four categories of variables can be compared: climate, geomorphology, lithology, and land cover. Sources of gridded dataset and extraction of each basin-scale variable (*n* = 20) are described in Methods.

**Table 1:** Range of each environmental control variable in the compiled dataset.

Category	Variable^a^	Max.	Min.	Mean	Standard deviation
Climate	Mean annual temperature (^o^C)	28.1	−12.5	12.8	8.0
	Temperature seasonality^b^ (^o^C)	14.8	0.2	6.5	2.7
	Mean annual precipitation (m/yr)	3.8	0.1	0.9	0.6
	Precipitation seasonality^c^ (%)	149%	6%	42%	27%
	Actual evapotranspiration (m/yr)	1.6	0.1	0.6	0.3
Geomorphology	Modeled sediment yield (t/km^2^/yr)	3.6E+03	2.2E+00	1.9E+02	2.9E+02
	Flow length (km)	1.4E+03	7.4E-01	1.6E+02	1.8E+02
	Drainage area (km^2^)	1.0E+05	1.5E-01	6.2E+03	1.3E+04
	Mean elevation (m)	3.9E+03	4.1E+00	5.3E+02	5.7E+02
	Mean local slope (^o^)	35.1	0.4	6.1	6.1
	Upland hillslope regolith thickness (m)	50.0	0.0	17.2	8.5
	Upland hillslope soil thickness (m)	1.5	0.1	0.8	0.2
Lithology	Acidic-intermediate rock area (%)	100%	0%	10%	24%
	Basic rock area (%)	100%	0%	4%	16%
	Clastic sedimentary rock area (%)	100%	0%	57%	42%
	Carbonate rock area (%)	100%	0%	15%	31%
	Metamorphic rock area (%)	100%	0%	14%	30%
Land cover	Vegetation cover area (%)	100%	0%	94%	16%
	Tree cover area (%)	100%	0%	38%	35%
	Ice cover area (%)	100%	0%	1%	8%

^a^Dataset of all the variables for each river basin are reported in Supplementary Data 2.

^b^Temperature seasonality is calculated as the standard deviation of monthly temperature.

^c^Precipitation seasonality is calculated as the coefficient of variation of monthly precipitation.

**Fig. 3:**
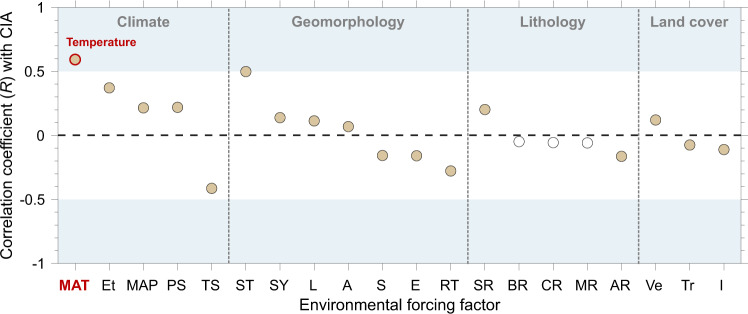
Correlation coefficients (*R*) between each environmental forcing factor (*n* = 20) and CIA. Correlations with *p* value of >0.001 are shown as open symbols. The order of forcing factors in each category is sorted by correlation coefficient with CIA. Negative values mean a negative correlation. Most of |*R*| are lower than 0.5, indicating a subordinate control of these factors on weathering intensity over a global scale. Mean annual temperature (MAT) is the only factor with |*R* | of >0.5. The correlation coefficients (*R*) with WIP are provided in Supplementary Fig. 2. Climatic metric: MAT-mean annual temperature (^o^C), Et-actual evapotranspiration (m/yr), MAP-mean annual precipitation (m/yr), PS-precipitation seasonality (coefficient of variation, %), TS-temperature seasonality (standard deviation, °C); geomorphic metric: ST-upland hillslope soil thickness (m), SY-modeled sediment yield (t/km^2^/yr) using BQART model^29^, L-flow length (km), A-drainage area (km^2^), S-mean local slope (^o^), E-mean elevation (m), RT-upland hillslope regolith thickness (m); lithological metric: SR-clastic sedimentary rock area (%), BR-basic rock area (%), CR-carbonate rock area (%), MR-metamorphic rock area (%), AR-acidic-intermediate rock area (%); land cover metric: Ve-vegetation cover area (%), Tr-tree cover area (%), I-ice and snow cover area (%).

To identify key variables governing weathering indices, we assess correlation coefficients (*R*) between each environmental forcing factor and CIA (Fig. 3). Rather weak correlations exist between weathering index and land cover factors (|*R* | ≤ 0.12) or lithological factors (|*R* | ≤ 0.20). The enhancement of silicate weathering by plants is limited to certain environmental conditions related to soil matrix and generation of organic matters^25^, and weathering intensity can even be decoupled from plant productivity^26^. Ice cover is spatially limited to high-latitude and high-altitude regions with only local impact. Effect of source rocks likely controls weathering indices in coarse-grained sediments (e.g., sand) given the enrichment of unaltered primary minerals, while overprints of weathering processes dominate in fine-grained sediments enriched in clay minerals^27^. Likewise, similar variability in mobile elements can be found in fine-grained topsoil from different rock types^22^. To illustrate the lithological effect more intuitively, we modeled the CIA of source rock for each sampling basin using the areal percentage of each rock type and their typical chemical compositions^28^. The correlation between sediment CIA and modeled CIA of the source rock is positive but rather weak (*R* = 0.21, Supplementary Fig. 4), suggesting a potential, but only subordinate, lithological control of weathering index.

Basin-scale geomorphic characteristics such as drainage area, flow length, mean elevation, mean local slope, and regolith thickness show no strong correlations (|*R* | ≤ 0.28) with CIA. The correlation with modeled sediment yield from BQART model^29^ (Methods) is also poor (Fig. 3). The relationship between physical erosion and chemical weathering can vary depending on weathering regimes (supply-limited versus weathering-limited)^8,30,31^, and the lack of correlation between sediment yield and weathering intensity here hints at a decoupling between both factors in our compiled dataset. By contrast, a moderate (|*R* | = 0.49, *p* < 0.001) positive correlation is observed between CIA and soil thickness. Globally, a higher weathering intensity corresponds to thicker soils, consistent with the notion that thick soil profiles are enriched in secondary clay minerals^32^.

As to climatic forcing, much current debate surrounds the response of weathering indices to individual factors, that are, temperature versus precipitation. There are three widely used mechanisms including temperature control^33^, precipitation control^34,35^, and climate (temperature and precipitation) control^36^. We attribute such discrepancy in data interpretation to either (1) covariation of both factors or (2) much smaller variability in one factor compared to the other in local- and regional- scale studies. Here we have the possibility to test the three mechanisms above given their poor correlation in our compiled dataset (*R* = 0.15) and the large gradient in each climatic factor (mean annual precipitation: 0.1 to 3.8 m/yr; mean annual temperature: −12.5 to 28.1 °C, Table 1).

**Fig. 4:**
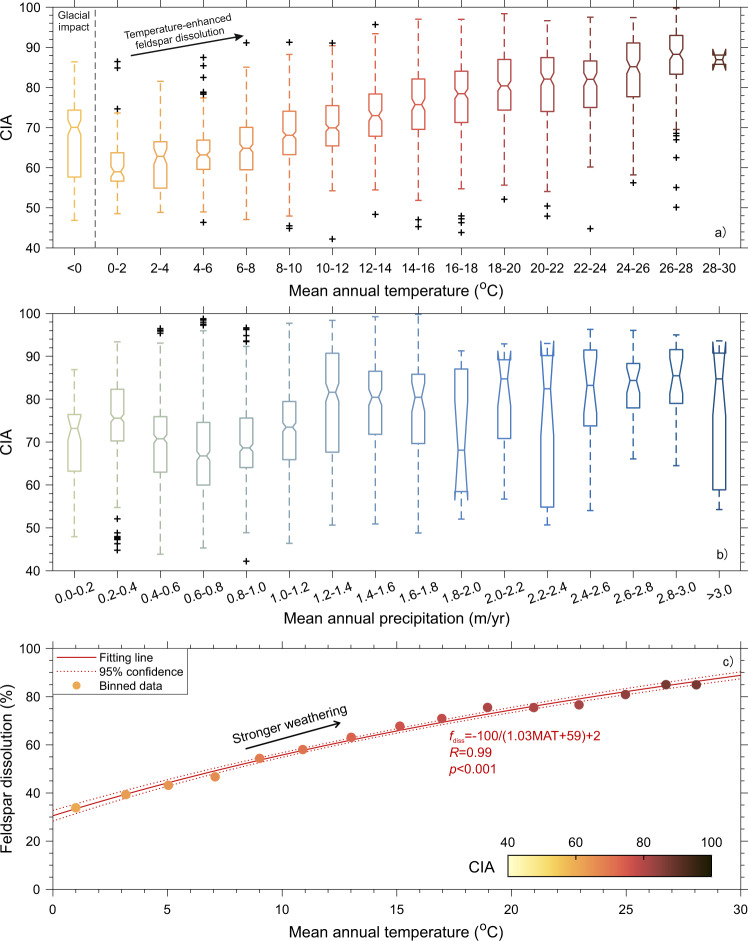
Control of temperature versus precipitation on CIA. **a**, **b** Boxplots of CIA grouped by zones of MAT (**a**, interval of 2 °C) and MAP (**b**, interval of 0.2 m/yr). The notch of each box displays the variability of the median: if the notches of both boxes do not overlap, their medians are significantly different at the 5% level. The box limits are 25th and 75th percentiles, the whiskers represent the 1.5× interquartile range, and the “+” symbols are outliers. CIA increases monotonically with MAT and shows a more complicated pattern with MAP. The patterns of WIP (Supplementary Fig. 3) are similar to those of CIA shown here. **c** Empirical relationship between MAT and percentage of feldspar dissolved (*f*_diss_, %) converted from average sediment CIA (Methods) binned by temperature (an interval of 2 °C, MAT >0 °C, Supplementary Fig. 6). The non-negligible *f*_diss_ under low MAT (~0 °C) might be partly contributed by sedimentary rock outcrops that have been weathered before exposure. However, the impact of such *f*_diss_ baseline in a specific region can be largely reduced when calculating relative changes of *f*_diss_ with MAT.

Temperature control of weathering intensity is evident as the correlation coefficient between mean annual temperature (MAT) and CIA (*R* = 0.60) is the highest among all the variables investigated (Fig. 3), while the correlation with precipitation (MAP) is poor (0.21). Furthermore, when grouping chemical index data by each climatic variable to minimize the potential effects of other factors (Fig. 4a, b), we find that CIA can either increase or decrease with MAP at a certain interval (Fig. 4b). CIA decreases with MAP at an interval of 0.2–0.8 m/yr and increases with MAP at 0.8–1.4 m/yr, and then keeps relatively constant at a MAP of >2 m/yr (Fig. 4b). The correlation coefficients between MAP and weathering indices are mostly low even within a narrow range of MAT (Supplementary Table 1), indicating that such weak precipitation control can not be explained by temperature variability. We interpret the variable correlations as a competing effect: precipitation can increase weathering intensity by supplying fluid for chemical reaction^11^ or weaken it by driving erosion^37^, such as storm-triggered mass wasting^38^, and then decreasing soil residence time for reaction. The operation of either effect can dominate on a local scale under different environmental conditions^11,37^. By contrast, a monotonic increase of CIA with MAT (MAT >0 °C, Fig. 4a) is explained by enhancement of chemical weathering reaction under high temperature^9,39^. At MAT below 0 °C, we attribute the relatively high CIA (~70) to glacial processes, which can either drive the rapid supply of fresh materials for weathering via frost action and abrasion and formation of Fe-/Mg- rich secondary minerals^40,41^ such as chlorite (CIA of ~100^14^) or result in multiple sediment exposure-burial cycles^42^.

## Discussion

Based on the weathering index dataset of the highest spatial resolution ever, we above revisited the effects of individual environmental variables on silicate weathering intensity. We emphasize the primary control of surface temperature over the continental scale, although, in a few settings dominated by e.g., rainfall-triggered mass wasting or glacial processes, multiple other factors may also play some role and cause the deviation of CIA from the overall trend (Fig. 4a). In general, the genetic link between MAT and CIA sheds new light on the promising application of CIA for paleoclimate reconstruction. CIA in siliciclastic sediment archives can record distinct glacial-interglacial variability^43^, but such temporal change is not readily interpreted as a paleo-temperature signal in a quantitative way^24^. Here we establish an empirical relation between MAT and CIA using our compiled CIA data binned by MAT intervals (2 °C each). The binning strategy is to minimize the CIA variability caused by factors other than the temperature at a regional scale (Fig. 4a). The fitting between MAT and CIA results in a linear equation (Supplementary Fig. 6). We consider it to be valid over large spatial or temporal scales given the strong correlation (*R* = 0.99) and its insensitivity to variability in chosen grain sizes, mineralogical sources, and dominant provenance lithology (Methods, Supplementary Table 2).

To first test the utility of the MAT–CIA equation in modern times, we derive a global-average CIA by substituting global mean surface air temperature (GSAT, 14 °C)^44^ into this equation. The resulting CIA (73.5) is consistent with previous global-average estimates (71.6–75.5)^15,17,45^. When applying the MAT–CIA equation to quantitatively estimate paleo-MAT at a regional scale, it is worth noting that the CIA record could be affected by multiple non-climatic factors that may introduce a bias into the paleo-MAT reconstruction. First, changes in grain size (sand vs. silt-clay) and provenance in the given sedimentary archive can alter the CIA values^46,47^. In addition, spatial variability of other environmental variables such as topography and lithology may cause several-unit variations of CIA at a given MAT as observed in the modern sediment dataset (Fig. 4a). Hence, in order to extract accurate information on paleo-MAT from sedimentary records, the factors mentioned above should be well-constrained at the studied interval. As such, several prerequisites on sedimentary archives need to be met for applying the MAT–CIA equation: (1) archives are dominated by clay- and silt-sized sediments to reduce mineralogical differentiation; (2) the change in sediment provenance is minor based on constraints from independent provenance proxy; (3) the observation time interval should be at least 10^3^–10^4^ years for a sufficient response of weathering to temperature changes^43,48^. Furthermore, although the site-specific CIA baseline, that is, the MAT–CIA relation intercept, may vary across diverse locations depending on e.g., regional geology, its impact is likely relatively stable over time in one sedimentary record without major provenance change. Hence, (4) such impact can be largely canceled out by calculating relative changes in CIA (∆CIA). Accordingly, we can only estimate relative changes in MAT (i.e., ∆MAT = ∆CIA/1.02, Supplementary Fig. 6) rather than absolute values.

**Fig. 5:**
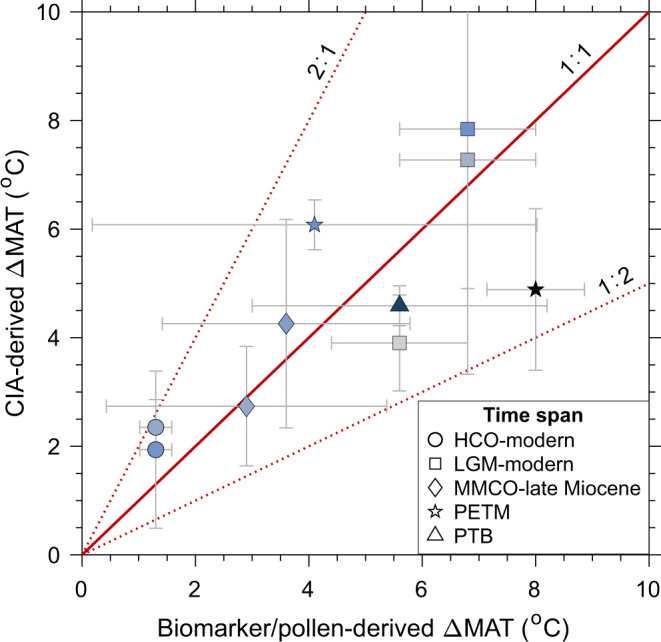
Reconstruction of regional paleo-temperature changes (∆MAT) for selected climatic events. The comparison between two methods, that are, MAT–CIA relation (this study) and biomarker or pollen records (from literature) are shown here. The events include Holocene Climatic Optimum (HCO), Last Glacial Maximum (LGM), Middle Miocene Climate Optimum (MMCO), Paleocene–Eocene thermal maximum (PETM), and Permian–Triassic boundary (PTB). The symbol color indicates the sampling latitude of sedimentary CIA records: a darker color corresponds to a higher latitude (range: 8–78°). The error bars represent one standard deviation. The relative uncertainty of ∆MAT estimated from paleo-CIA records could even be lower compared to the other method especially in pre-Quaternary events. We note that terrestrial paleo-temperature reconstructed by biomarker/pollen records are scarce and rarely measured at the same location as sediment CIA. Hence, we tried to find synchronous biomarker/pollen records at a latitude similar to the sediment source region. Distributions of ∆MAT derived from both methods are indistinguishable based on a paired *t*-test (*p* > 0.05). Detailed information including references on sediment cores and paleo-temperature records is provided in Supplementary Data 3.

We then assess the utility of CIA-derived paleo-temperature by comparison with synchronous regional paleo-temperature records reconstructed by an independent approach (e.g., biomarker). We selected literature paleo-CIA records (*n* = 10) that cover well-known warming/cooling events, in order to focus on the control of temperature and minimize potential effects of other factors. Selected events include the Permian–Triassic boundary (PTB), Paleocene–Eocene thermal maximum (PETM), Middle Miocene Climate Optimum (MMCO), Last Glacial Maximum (LGM), and Holocene Climatic Optimum (HCO) (Supplementary Data 3). With regional paleo-temperature changes (∆MAT) of up to 8 °C in each case, reconstructions from CIA and biomarker/pollen records show an offset varying from <1 to 3 °C and generally agree within uncertainty (Fig. 5). The offset is surprisingly small considering that non-climatic factors such as provenance changes can shift CIA values to such magnitude even over a seasonal scale^49^. Furthermore, distributions of ∆MAT derived from both methods (Fig. 5) are indistinguishable based on a paired *t*-test (*p* > 0.05). We admit that such preliminary evaluation may need further supporting evidence from more fine-grained sedimentary records with well-constrained information on regional paleo-MAT and provenance. Nevertheless, it demonstrates the great potential of the MAT–CIA equation for providing the first-order estimate on paleo-temperature changes with an uncertainty of ~3 °C, especially in the deep time (10^8^–10^9^ years) when other paleo-temperature proxies such as biomarkers are not readily applicable.

The quantitative relationship between surface temperature and silicate weathering intensity over large spatio-temporal scales also offers valuable insights into the climate-weathering feedback. To link temperature with weathering-driven CO_2_ consumption using CIA, we first convert the binned MAT–CIA relation to a relation between MAT and percentage of feldspar dissolution (*f*_diss_ = −100/(1.03MAT + 59) + 2, Fig. 4c) based on the stoichiometric link of *f*_diss_ with CIA (Methods). There is a nonlinear response of feldspar dissolution to temperature, that is, a weaker response at higher MAT. We attribute such non-linearity to depletion of plagioclase and thus dominance of orthoclase weathering at the advanced stage of weathering under high MAT^18^ (Supplementary Fig. 7). Orthoclase with a lower dissolution rate constant and lower activation energy of reaction^7^ may have a weaker temperature dependence during weathering. The changes in mineral types available for weathering with temperature support the hypothesis proposed by recent results of carbon cycle modeling^1^: with progressive cooling during the late Cenozoic, the land surface reactivity is suggested to increase for reconciling different paleo-weathering records, that is, a higher ratio of plagioclase supply (more reactive) to orthoclase supply for weathering according to our scenario.

**Fig. 6:**
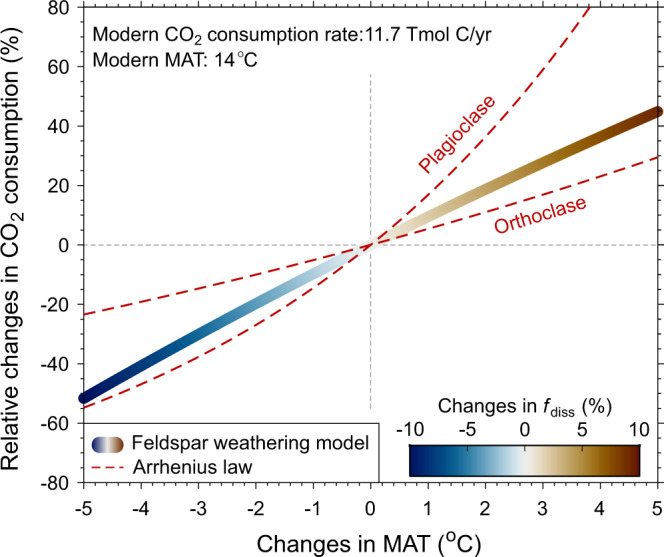
Response of modeled silicate weathering-driven CO_2_ consumption to changes in MAT. The modeled link is derived from the feldspar weathering model based on MAT-*f*_diss_ equation in Fig. 4c. The changes in both MAT and CO_2_ consumption are relative to the modern conditions. Note that changes in CO_2_ consumption here are estimated from Ca, Na, and K only (mobile elements in feldspar) without Mg, and thus represent a conservative estimate. First-order estimates on the temperature dependence of weathering reactions by the Arrhenius law are shown for comparison. The range of activation energy used is 36 kJ/mol (orthoclase) to 107 kJ/mol (plagioclase)^70^. Details on the feldspar weathering model are described in Methods.

To quantify CO_2_ consumption via silicate weathering, previous global carbon cycle models apply theoretical equations based on weathering reaction kinetics^5,48^, but such equations are rarely validated by large-scale field observations. Here we propose to implement our MAT-*f*_diss_ equation into such models as an alternative empirical approach. For example, our feldspar weathering model built on this equation and feldspar hydrolysis reactions (Fig. 6, Methods) suggests that transient global CO_2_ consumption resulting from silicate weathering can increase by 28% for a temperature increase of 3 °C relative to the modern (e.g., a global warming event). Such estimate falls within the range predicted by the Arrhenius law on the temperature dependence of weathering reactions (17–59%, Methods).

In conclusion, the nonlinear forcing of surface temperature on feldspar dissolution (constrained by CIA) is caused by depletion of plagioclase relative to orthoclase at high temperatures. In another word, the surface temperature decrease could be accompanied by a higher proportion of more reactive plagioclase available for weathering, supporting the hypothesis that land surface reactivity has increased during the late Cenozoic cooling. We also propose a first-order quantitative relationship between surface temperature, feldspar dissolution, and CO_2_ consumption that will be of great potential for deep-time temperature reconstruction and carbon cycle modeling.

## Methods

### Sample selection

We aimed to compile a geochemical dataset of all river sediment samples from peer-reviewed papers and publicly available reports based on the following criteria. (1) Only fine-grained samples were compiled in this study to minimize the effect of mineralogical differentiation and contribution of unaltered parent materials. Selected samples include surface suspended particulate matter (SPM), bedload of silt-/clay- size (based on sample description) and bedload sieved to a narrow grain-size window (e.g., <63, <32, and <2 μm). (2) Concentrations of major elements including Al, Ca, Na, K, and Mg were reported for CIA and WIP calculation. (3) Sediments were fully decomposed using multiple-acid digestion method (including HF) or fusion with e.g., LiBO_2_ prior to measurements, or samples were processed by X-ray fluorescence; samples only leached by HNO_3_ or HCl were not eligible. (4) Samples with a molar ratio of K to Al higher than 1 were excluded in this study as such ratio is even higher than K-feldspar (K/Al = 1) and is not composed of common silicate minerals. We finally compiled 3828 samples that met all the criteria above. A full reference list of the compiled dataset is provided in Supplementary Information. It is worth mentioning that large major element dataset from certain regions were sourced from geochemical baseline database including the NAWQA program in the conterminous US^50^, the AGDB2 program in Alaska (US)^51^, FOREGS program in Europe^52^, and NGSA program in Australia^53^.

### Feldspar hydrolysis reactions and chemical weathering index

The weathering process can be viewed as a transformation of primary minerals (feldspar) to secondary products (clay minerals) by neutralizing carbonic acid derived from the atmosphere^18^. Feldspar dissolution is the most common silicate weathering reaction given its high abundance in the upper continental crust. There are three major types of feldspar including potassium feldspar (KAlSi_3_O_8_, orthoclase), albite, and anorthite (NaAlSi_3_O_8_ and CaAl_2_Si_2_O_8_, plagioclase). Their hydrolysis reactions can be simplified as follows^54^:

1 $$2{{{{{{\rm{KAlSi}}}}}}}_{3}{{{{{{\rm{O}}}}}}}_{8} 	+2{{{{{{\rm{CO}}}}}}}_{2}+11{{{{{{\rm{H}}}}}}}_{2}{{{{{\rm{O}}}}}}\to {{{{{{\rm{Al}}}}}}}_{2}{{{{{{\rm{Si}}}}}}}_{2}{{{{{{\rm{O}}}}}}}_{5}{({{{{{\rm{OH}}}}}})}_{4}\,({{{{{\rm{Kaolinite}}}}}})+2{{{{{{\rm{K}}}}}}}^{+}\\ 	 +2{{{{{{\rm{HCO}}}}}}}_{3}^{-}+4{{{{{{\rm{H}}}}}}}_{4}{{{{{{\rm{SiO}}}}}}}_{4}$$

2 $$2{{{{{{\rm{NaAlSi}}}}}}}_{3}{{{{{{\rm{O}}}}}}}_{8}+2{{{{{{\rm{CO}}}}}}}_{2}+11{{{{{{\rm{H}}}}}}}_{2}{{{{{\rm{O}}}}}}\to {{{{{{\rm{Al}}}}}}}_{2}{{{{{{\rm{Si}}}}}}}_{2}{{{{{{\rm{O}}}}}}}_{5}{({{{{{\rm{OH}}}}}})}_{4}+2{{{{{{\rm{Na}}}}}}}^{+}+2{{{{{{\rm{HCO}}}}}}}_{3}^{-}+4{{{{{{\rm{H}}}}}}}_{4}{{{{{{\rm{SiO}}}}}}}_{4}$$

3 $${{{{{{\rm{CaAl}}}}}}}_{2}{{{{{{\rm{Si}}}}}}}_{2}{{{{{{\rm{O}}}}}}}_{8}+2{{{{{{\rm{CO}}}}}}}_{2}+3{{{{{{\rm{H}}}}}}}_{2}{{{{{\rm{O}}}}}}\to {{{{{{\rm{Al}}}}}}}_{2}{{{{{{\rm{Si}}}}}}}_{2}{{{{{{\rm{O}}}}}}}_{5}{({{{{{\rm{OH}}}}}})}_{4}+{{{{{{\rm{Ca}}}}}}}^{2+}+2{{{{{{\rm{HCO}}}}}}}_{3}^{-}$$

Chemical index of alteration (CIA)^14^ and weathering index of Parker (WIP)^16^ directly indicate the dissolved loss of mobile cations and track the feldspar hydrolysis reaction to some extent. CIA is calculated as the molar ratio of immobile metal oxide to mobile metal oxides:

4 $${{{{{\rm{CIA}}}}}}=\frac{{{{{{{\rm{Al}}}}}}}_{2}{{{{{{\rm{O}}}}}}}_{3}}{{{{{{{\rm{Al}}}}}}}_{2}{{{{{{\rm{O}}}}}}}_{3}+{{{{{{\rm{CaO}}}}}}}^{\ast }+{{{{{{\rm{Na}}}}}}}_{2}{{{{{\rm{O}}}}}}+{{{{{{\rm{K}}}}}}}_{2}{{{{{\rm{O}}}}}}}\times 100$$

Where CaO* is the CaO incorporated in the silicate fraction. We followed the approach proposed by McLennan^15^ to correct for Ca contribution from non-silicate fractions. CaO was first corrected for phosphate by subtracting 10/3 of P_2_O_5_ from CaO where available. If the remaining moles were less than that of Na_2_O, the CaO value was adopted. Otherwise, calcium carbonate contribution may be significant and CaO* was assumed to be equivalent to Na_2_O for CIA calculation, as this Ca/Na ratio is reasonable in silicate minerals. McLennan^15^ noted that such treatment may slightly underestimate CIA values if weathering loss of Ca is more rapid than that of Na; but in catchments dominated by basic rock and weak weathering, such treatment may slightly overestimate CIA because a certain proportion of Ca is derived from Ca-silicates other than feldspar. Since a uniform data-processing procedure is a prerequisite for a global-scale comparison, we sticked to McLennan’s approach and assumed that a minor bias in CIA calculation on a local scale is much smaller than the variability caused by each forcing factor^49^ and can be averaged out over a global scale.

WIP is calculated as the molar proportion of mobile elements and weighted by the bond strength of each element with oxygen as a measure of susceptibility to weathering:

5 $${{{{{\rm{WIP}}}}}}=\left(\frac{2{{{{{{\rm{Na}}}}}}}_{2}{{{{{\rm{O}}}}}}}{0.35}+\frac{{{{{{\rm{MgO}}}}}}}{0.90}+\frac{2{{{{{{\rm{K}}}}}}}_{2}{{{{{\rm{O}}}}}}}{0.25}+\frac{{{{{{{\rm{CaO}}}}}}}^{\ast }}{0.70}\right)\times 100$$

where CaO* is the silicate-bound CaO same as that in CIA calculation.

### Rationale for focus on CIA-WIP opposed to other weathering indices

To delineate the global pattern of silicate weathering intensity, several requirements on weathering index need to be met: (1) applicability to diverse rock types and large dataset available across the globe, (2) incorporation of elements with a range of mobility during weathering, and (3) monotonic response to silicate weathering^55^. Both CIA and WIP meet such requirements but many other weathering proxies do not. Alpha (*α*) indices^19^ using a ratio of one immobile element to one mobile element can only track the mobility of a specific element and a certain stage of weathering. Isotopic tools like Li isotopes and K isotopes are limited by their small size of the dataset available due to strict requirements on precise measurement, and they either do not monotonically increase with weathering intensity^56^ or can show a small variability during weathering processes^57^. The rate ratio of weathering to denudation (*W*/*D*) based on measurements of dissolved and solid load^58^ is widely used and can be instructive for understanding weathering kinetics. However, its integration timescale is too short (10^0^–10^1^ yr). As such, this proxy may be subject to anthropogenic impact and can not be applied in the geological past for reconstructing paleo-signal of weathering and thus climate.

### Geospatial analysis: quantification of environmental parameters

We used Matlab-based software TopoToolBox 2 for digital terrain analysis and gridded dataset processing^59^. The upstream basin outline of each sample was calculated using Shuttle Radar Topography Mission (SRTM) 90-m resolution Digital Elevation Model (DEM) (https://opentopography.org/). At latitudes of >~60° where no SRTM data is available, alternative sources of topographic data were adopted from http://viewfinderpanoramas.org/dem3.html. All the basin-averaged environmental control variables including climatic, geomorphic, lithological, and land cover metrics were extracted using the corresponding basin outline and based on a publicly available gridded dataset.

(1) Climatic metric: mean annual temperature (°C, MAT), temperature seasonality (standard deviation, °C), mean annual precipitation (m/yr, MAP), and precipitation seasonality (coefficient of variation, %) were calculated from gridded climate dataset (WorldClim 2^60^) with ~1 km resolution, and actual evapotranspiration was calculated from Global High-Resolution Soil-Water Balance dataset with ~1 km resolution^61^. (2) Geomorphic metric: flow length (km), drainage area (km^2^), mean local slope (°), mean elevation (m), and maximum relief (m) were calculated from SRTM 90-m resolution DEM using TopoToolBox, and upland hillslope soil and regolith thickness (m) were calculated from a gridded global dataset with ~1 km resolution^62^. The regolith includes soil and weathered bedrock and is estimated as the depth to the permanent water table^62^. (3) Lithological metric: the areal percentage of each major rock type including acidic-intermediate rock, basic rock, clastic sedimentary rock, carbonate rock, and metamorphic rock were calculated from the global lithological map database GLiM, which represents the rock types of the Earth surface with ~1.23 × 10^6^ polygons^63^ but might be less precise in locations with large lithological heterogeneity; the rock erodibility index was calculated from a gridded dataset with ~0.1 degree resolution^64^. (4) Land cover metric: the areal percentage of vegetation cover (sum of all vegetation types), tree cover (sum of all tree types), and ice-snow cover were calculated from a gridded dataset (GLC2000) with ~1 km resolution^65^. All the original gridded datasets were downscaled to a resolution same to DEM (90 m) for metric calculation.

### Criteria for extracting environmental control variables

We only extracted basin-scale environmental control variables for a sub-sample set compiled in this study based on criteria as follows. (1) For samples collected at the same location but sieved to different grain sizes (e.g., <2 μm vs. <63 μm) or collected using different approaches (e.g., bedload vs. SPM), we only chose the fraction of the finest grain size for environmental variable extraction. (2) The geospatial analysis was only performed on samples with an upstream area of <10^5^ km^2^, that is, small- and median-sized catchment. Large rivers (e.g., >10^5^ km^2^) drain a large gradient of each environmental variable, and thus their basin-averaged values can be well homogenized and it is difficult to distinguish the effect of one factor from the others. (3) The geospatial analysis requires latitude and longitude of one sample to delineate the outline of its upstream basin, and thus samples without location information could not be used. For some downstream/outlet samples we did estimate such information using Google Earth if the original study provided a geographical map. We finally processed 2989 samples that met all the criteria above.

### Computation of sediment yield

We applied the BQART model^29^ to derive a sediment yield of each studied basin for comparison with weathering indices, as most of our compiled dataset were from small- and median-sized basins with no hydrological information available. The BQART model has been applied in catchments with a wide range of sediment yields (10^0^–10^4^ t/km^2^/yr)^29^ and only requires inputs of topographic, lithological, and climatic parameters that can be extracted from the available gridded dataset. The model equations are as follows:

6 $${Y}_{s}=w\times B\times {Q}^{0.31}\times {A}^{-0.5}\times {R}_{\max }\times {{{{{\rm{MAT}}}}}}\times {10}^{6}({{{{{\rm{MAT}}}}}}\ge {2}\;\deg\! {{{{{\rm{C}}}}}})$$

7 $${Y}_{s}=2w\times B\times {Q}^{0.31}\times {A}^{-0.5}\times {R}_{\max }\times {10}^{6}({{{{{\rm{MAT}}}}}} < {2}\;\deg\! {{{{{\rm{C}}}}}})$$

where *Y*_s_ is sediment yield in t/km^2^/yr, *ω* is a coefficient as 0.0006 Mt/yr, *A* is drainage area in km^2^, *R*_max_ is maximum relief in km and MAT is mean annual temperature in °C. *Q* is water discharge in km^3^/yr and calculated by multiplying runoff (mean annual precipitation minus actual evapotranspiration) by drainage area. *B* is a coefficient accounting for geological and human factors and calculated as:

8 $$B=(1+9{A}_{g})\times {L}_{r}\times (1-{T}_{E})\times {E}_{h}$$

where *A*_g_ is the areal percentage (range of 0–1) of ice and snow cover and indicates glacier erosion, *L*_r_ is the basin-wide rock erodibility index, *T*_E_ is the sediment trapping efficiency and set to 0 in this case, and *E*_h_ is a human-influenced soil erosion factor and set to 1 as weathering indices commonly integrate over a timescale longer than that of human impact.

All the input parameters were derived from the geospatial analysis mentioned above. Dataset of input parameters and modeled sediment yield are reported in Supplementary Data 2.

### Sensitivity test on MAT–CIA relation

To test if the correlation between MAT and CIA persists for sediments of different features, we performed a sensitivity test on the MAT–CIA relationship by deriving such fitting equations separately (1) for sediments of different grain sizes, that is, SPM and clay fraction vs. bedload of <63 μm, (2) for sediments weathered from different mineralogical sources (indicated by different slopes of CIA-WIP plot, Supplementary Fig. 5), and (3) for catchments characterized by different lithologies. For each scenario, the relation slopes and correlation coefficients of most fitting lines agree within uncertainty (Supplementary Table 2), suggesting the robustness of the MAT–CIA relationship to a large extent.

### Stoichiometric relation between feldspar dissolution and CIA

We assumed that the hydrolysis reaction, that is, feldspar dissolution and formation of kaolinite, is the dominant reaction that controls variability in fine-grained CIA over a global scale^14^. As such, we could link CIA to the molar ratio of kaolinite to feldspar in a sediment sample based on the stoichiometry of feldspar hydrolysis reactions (eqs. 1–3), and converted CIA to the proportion of feldspar dissolution (*f*_diss_):

9 $${f}_{{{{{{\rm{diss}}}}}}}=-100/{{{{{\rm{CIA}}}}}}+2$$

Note that this CIA-*f*_diss_ relation is consistent for all types of feldspar (Supplementary Fig. 5b).

### Feldspar weathering model based on MAT-*f*_diss_ equation

We established a simplified relation between the proportion of feldspar dissolution (*f*_diss_) and weathering-driven atmospheric CO_2_ consumption using the following procedure.

(1) We first estimated the relative release rate of Ca, Na, and K during feldspar dissolution as the feldspar is characterized by three endmembers (anorthite, albite, and K-feldspar) of different chemical compositions. To do so we multiplied the molar proportion of each kind of feldspar in the average upper continental crust^18^ by the release rate constant of each metal in the corresponding feldspar determined from dissolution kinetic experiments^18^. We then obtain the ratio of relative release rates as 0.32:0.51:0.17 (*R*_Ca_:*R*_Na_:*R*_K_). Such rate ratios calculated using kinetic experiment data reproduced the weathering trend (changes in the relative proportion of each metal) in some soil profiles^18^ and may be reasonable estimates here. (2) We estimated the global annual export flux of feldspar (*F*_fs_) by multiplying the percentage of feldspar in the upper continental crust (53% in volume)^18^ by the global annual riverine flux (22.8 Gt/yr^58^, assumed as a constant here), and *F*_fs_ = 4.44 × 10^13 ^mol/yr. (3) We calculated changes in the flux of each released cation (∆*F*_cation_, mol/yr) relative to the modern based on changes in *f*_diss_ (∆*f*_diss_, dependent on MAT):

10 $$\varDelta {F}_{{{{{{\rm{Ca}}}}}}}={F}_{{{{{{\rm{fs}}}}}}}\times \varDelta {f}_{{{{{{\rm{diss}}}}}}}\times {R}_{{{{{{\rm{Ca}}}}}}}$$

11 $$\varDelta {F}_{{{{{{\rm{Na}}}}}}}={F}_{{{{{{\rm{fs}}}}}}}\times \varDelta {f}_{{{{{{\rm{diss}}}}}}}\times {R}_{{{{{{\rm{Na}}}}}}}$$

12 $$\varDelta {F}_{{{{{{\rm{K}}}}}}}={F}_{{{{{{\rm{fs}}}}}}}\times \varDelta {f}_{{{{{{\rm{diss}}}}}}}\times {R}_{{{{{{\rm{K}}}}}}}$$

(4) We converted each ∆*F*_cation_ above to changes in transient CO_2_ consumption rate (mol C/yr). Specifically, feldspar hydrolysis reactions (eqs. 1–3) indicate the alkalinity supplied by feldspar weathering and delivered by e.g., rivers: release of 1 mol Ca consumes 2 mol CO_2_ and release of 1 mol Na or K consumes 1 mol of CO_2_. (5) The change in CO_2_ consumption rate was then normalized to the modern global CO_2_ consumption rate by silicate weathering (1.17 × 10^13^ mol C/yr)^66^. The modeling results are shown in Fig. 6. An implicit assumption here is that the flux of feldspar is mainly exported in the fine-grained form (<63 μm) as our compiled dataset. If the contribution of coarse-grained bedload sediments (sand-gravel) to the total sediment load is significant, our estimate on CO_2_ consumption could be an upper-bound limit. Nevertheless, such coarse sediment contribution and its bias on our calculation might be subordinate: (1) the suspended load may dominate in most of median- and large-sized catchments as its proportion increase with drainage area to some extent (e.g. >80% in basins of >10^3^ km^2^ based on an empirical relationship)^67^; (2) the fine fraction (clay-silt) can even prevail in the suspended load of small fast-eroding mountainous catchments during the flood events^68^.

For comparison purposes, the Arrhenius equation that describes the temperature dependence of mineral dissolution rates was also applied to estimate relative changes in silicate weathering fluxes^69^:

13 $$\frac{{r}_{{{{{{\rm{T}}}}}}}}{{r}_{0}}={{{{{{\rm{e}}}}}}}^{\frac{{E}_{{{{{{\rm{a}}}}}}}}{{R}_{{{{{{\rm{g}}}}}}}}\times (\frac{1}{{T}_{0}}-\frac{1}{T})}$$

where *r*_T_ and *r*_0_ are the dissolution rates at temperature *T* (in K) and at reference temperature *T*_0_ (287.15 K or 14 °C^44^ in this case), respectively, *E*_a_ is the activation energy for the reaction (kJ/mol), and *R*_g_ is the gas constant (8.314 J/mol/K). We here adopted a range of *E*_a_ (36–107 kJ/mol) determined from orthoclase and plagioclase at near-neutral pH^70^, although discrepancies in *E*_a_ may exist in literature due to diverse experimental conditions such as solution chemistry^7^. In general, such a first-order estimate on temperature dependence is in agreement with our feldspar weathering model (Fig. 6). For example, *r*_T_/*r*_0_ can increase by 17–59% with an increase of MAT of 3 °C and our model result (28%) falls within this range. Furthermore, the concave shape of the MAT-CO_2_ consumption relation is different from the convex form of the exponential Arrhenius law (Fig. 6). One hypothesis for such difference is that *E*_a_ may vary with temperature in our compiled dataset across diverse geographic and lithological settings. With an increase in temperature, the mineral type available for weathering might shift from plagioclase to orthoclase and thus cause a decline in *E*_a_^7^.

## Supplementary information

Supplementary Information

Description of Additional Supplementary Files

Supplementary Data 1–3

## Data Availability

All supporting data in this study are compiled from literature and available in the cited references mentioned in Methods. The dataset (Supplementary Data 1–3) is also available in the Zenodo repository (10.5281/zenodo.6066701).

## Code availability

All gridded dataset are processed and analyzed by the Matlab-based software TopoToolBox 2 (Schwanghart and Scherler, 2014). A template of Matlab code (CARVE) for catchment-scale environmental variable extraction is available in the Zenodo repository (10.5281/zenodo.6067052).
