# Supplementary material for: A global temperature control of silicate weathering intensity
Source: Nat Commun. 2022 Apr 4;13:1781. doi: 10.1038/s41467-022-29415-0 (PMC8980099; doi:10.1038/s41467-022-29415-0)
Supplement: Supplementary file 1 — Supplementary Information [file 41467_2022_29415_MOESM1_ESM.pdf]

Supplementary Information for

A global temperature control of silicate weathering intensity

Kai Deng[1,2], Shouye Yang[1], Yulong Guo[1]

[1]State Key Laboratory of Marine Geology, Tongji University, 200092 Shanghai, China

[2]Institute of Geochemistry and Petrology, Department of Earth Sciences, ETH Zürich, Clausiusstrasse 25, 8092 Zürich, Switzerland

This document contains:

- Full reference list of compiled major element dataset
- Supplementary Figs. 1-7
- Supplementary Tables 1-2

Introduction

This supplementary document can be divided into four sections, including: 1) full reference list of compiled major element dataset, 2) spatial distribution of WIP and its controls (Supplementary Figs. 1-3), 3) effect of lithology, mineralogy, and sediment grain-size on weathering index (Supplementary Figs. 4-5), and 4) relationship between climatic factors and weathering index (Supplementary Figs. 6-7 and Supplementary Tables 1-2).

Part I Full reference list of compiled major element dataset

Below is the list of references containing 1) fine-grained modern sediments (*n*=3828) with major element dataset for CIA and WIP calculation (shown in Fig. 1) and 2) records of paleo-CIA and paleo-temperature (shown in Fig. 5).

1. Modern sediment dataset

1.1) Asia[1, 2, 3, 4, 5, 6, 7, 8, 9, 10, 11, 12, 13, 14, 15, 16, 17, 18, 19, 20, 21, 22]

1.2) Africa[23, 24, 25, 26, 27, 28]

1.3) Australia and New Zealand[29, 30, 31, 32, 33]

1.4) Europe[34, 35, 36, 37, 38, 39, 40, 41, 42]

1.5) North America[43, 44, 45, 46, 47, 48]

1.6) South America[49, 50, 51, 52]

1.7) Polar (Greenland and Iceland)[53, 54, 55]

2. Records of paleo-CIA and paleo-temperature

2.1) Paleo-CIA records[22, 56, 57, 58, 59, 60, 61, 62, 63, 64, 65]

2.2) Paleo-temperature records[66, 67, 68, 69, 70, 71]

1. Singh SK, France-Lanord C. Tracing the distribution of erosion in the Brahmaputra watershed from isotopic compositions of stream sediments. Earth Planet Sci Lett 202, 645-662 (2002).
2. Singh M, Sharma M, Tobschall HJ. Weathering of the Ganga alluvial plain, northern India: implications from fluvial geochemistry of the Gomati River. Appl Geochem 20, 1-21 (2005).
3. Chetelat B, Liu C-Q, Wang Q, Zhang G. Assessing the influence of lithology on weathering indices of Changjiang river sediments. Chem Geol 359, 108-115 (2013).
4. Pehlivan R. The effect of weathering in the Buyukmelen River basin on the geochemistry of suspended and bed sediments and the hyrogeochemical characteristics of river water, Duzce, Turkey. J Asian Earth Sci 39, 62-75 (2010).
5. Tanaka K, Watanabe N. Size distribution of alkali elements in riverbed sediment and its relevance to fractionation of alkali elements during chemical weathering. Chem Geol 411, 12-18 (2015).
6. Liu Z, et al. Source-to-sink transport processes of fluvial sediments in the South China Sea. Earth Sci Rev 153, 238-273 (2016).
7. Maharana C, Srivastava D, Tripathi JK. Geochemistry of sediments of the Peninsular rivers of the Ganga basin and its implication to weathering, sedimentary processes and provenance. Chem Geol 483, 1-20 (2018).
8. He J, Garzanti E, Dinis P, Yang S, Wang H. Provenance versus weathering control on sediment composition in tropical monsoonal climate (South China) - 1. Geochemistry and clay mineralogy. Chem Geol, 119860 (2020).

9. Chen J, Wang F. Chemical composition of river particulates in eastern China. GeoJournal 40, 31-37 (1996).
10. Garzanti E, et al. Mineralogical and chemical variability of fluvial sediments 2. Suspended-load silt (Ganga–Brahmaputra, Bangladesh). Earth Planet Sci Lett 302, 107-120 (2011).
11. Deng K, Yang S, von Blanckenburg F, Wittmann H. Denudation Rate Changes Along a Fast-Eroding Mountainous River With Slate Headwaters in Taiwan From 10Be (Meteoric)/9Be Ratios. Journal of Geophysical Research: Earth Surface 125, e2019JF005251 (2020).
12. Deng K, et al. Small dynamic mountainous rivers in Taiwan exhibit large sedimentary geochemical and provenance heterogeneity over multi-spatial scales. Earth Planet Sci Lett 505, 96-109 (2019).
13. Guo Y, Yang S, Su N, Li C, Yin P, Wang Z. Revisiting the effects of hydrodynamic sorting and sedimentary recycling on chemical weathering indices. Geochim Cosmochim Acta 227, 48-63 (2018).
14. Bi L, et al. Geochemistry of river-borne clays entering the East China Sea indicates two contrasting types of weathering and sediment transport processes. Geochemistry Geophysics Geosystems 16, 3034-3052 (2015).
15. Shao J, Yang S, Li C. Chemical indices (CIA and WIP) as proxies for integrated chemical weathering in China: inferences from analysis of fluvial sediments. Sediment Geol 265, 110-120 (2012).
16. Shevchenko VP, et al. On the elemental composition of suspended matter of the Severnaya Dvina River (White Sea region). Dokl Earth Sci 430, 228-234 (2010).
17. Ranasinghe PN, Fernando GWAR, Dissanayake CB, Rupasinghe MS. Stream sediment geochemistry of the Upper Mahaweli River Basin of Sri Lanka—Geological and environmental significance. J Geochem Explor 99, 1-28 (2008).
18. Gordeev VV, Rachold V, Vlasova IE. Geochemical behaviour of major and trace elements in suspended particulate material of the Irtysh river, the main tributary of the Ob river, Siberia. Appl Geochem 19, 593-610 (2004).
19. Rachold V. Major, Trace and Rare Earth Element Geochemistry of Suspended Particulate Material of East Siberian Rivers Draining to the Arctic Ocean. In: Land-Ocean Systems in the Siberian Arctic: Dynamics and History (eds Kassens H, et al.). Springer Berlin Heidelberg (1999).
20. Gaillardet J, Dupré B, Allègre CJ. Geochemistry of large river suspended sediments: silicate weathering or recycling tracer? Geochim Cosmochim Acta 63, 4037-4051 (1999).
21. Rachold V, Alabyan A, Hubberten HW, Korotaev VN, Zaitsev AA. Sediment transport to the Laptev Sea–hydrology and geochemistry of the Lena River. Polar Research 15, 183-196 (1996).
22. Lupker M, France-Lanord C, Galy V, Lavé J, Kudrass H. Increasing chemical weathering in the Himalayan system since the Last Glacial Maximum. Earth Planet Sci Lett 365, 243-252 (2013).

23. Garzanti E, Padoan M, Setti M, Najman Y, Peruta L, Villa IM. Weathering geochemistry and Sr-Nd fingerprints of equatorial upper Nile and Congo muds. Geochem Geophys Geosyst 14, 292-316 (2013).
24. Garzanti E, Padoan M, Setti M, López-Galindo A, Villa IM. Provenance versus weathering control on the composition of tropical river mud (southern Africa). Chem Geol 366, 61-74 (2014).
25. Schneider S, Hornung J, Hinderer M, Garzanti E. Petrography and geochemistry of modern river sediments in an equatorial environment (Rwenzori Mountains and Albertine rift, Uganda) — Implications for weathering and provenance. Sediment Geol 336, 106-119 (2016).
26. Dinis PA, Garzanti E, Hahn A, Vermeesch P, Cabral-Pinto M. Weathering indices as climate proxies. A step forward based on Congo and SW African river muds. Earth Sci Rev 201, 103039 (2020).
27. Compton JS, Maake L. Source of the suspended load of the upper Orange River, South Africa. S Afr J Geol 110, 339-348 (2007).
28. Chen Y, Hedding DW, Li X, Greyling AC, Li G. Weathering dynamics of Large Igneous Provinces (LIPs): A case study from the Lesotho Highlands. Earth Planet Sci Lett 530, 115871 (2020).
29. Kautz CQ, Martin CE. Chemical and physical weathering in New Zealand’s Southern Alps monitored by bedload sediment major element composition. Appl Geochem 22, 1715-1735 (2007).
30. de Caritat P, Cooper M. National Geochemical Survey of Australia: The Geochemical Atlas of Australia: Dataset. Geoscience Australia, Canberra, (2011).
31. Douglas GB, Ford PW, Palmer MR, Noble RM, Packett RJ, Krull ES. Fitzroy River Basin, Queensland, Australia. IV. Identification of flood sediment sources in the Fitzroy River. Environ Chem 5, 243-257 (2008).
32. Olley J, Caitcheon G. Major element chemistry of sediments from the Darling–Barwon river and its tributaries: implications for sediment and phosphorus sources. Hydrol Processes 14, 1159-1175 (2000).
33. Douglas GB, Hart BT, Beckett R, Gray CM, Oliver RL. Geochemistry of Suspended Particulate Matter (SPM) in the Murray-Darling River System: A Conceptual Isotopic/Geochemical Model for the Fractionation of Major, Trace and Rare Earth Elements. Aquat Geochem 5, 167-194 (1999).
34. Roy S, Gaillardet J, Allègre CJ. Geochemistry of dissolved and suspended loads of the Seine River, France: anthropogenic impact, carbonate and silicate weathering. Geochim Cosmochim Acta 63, 1277-1292 (1999).
35. Dannhaus N, Wittmann H, Krám P, Christl M, Von Blanckenburg F. Catchment-wide weathering and erosion rates of mafic, ultramafic, and granitic rock from cosmogenic meteoric 10Be/9Be ratios. Geochim Cosmochim Acta 222, 618-641 (2018).

36. Salminen R, Demetriades A, Reeder S. FOREGS-EuroGeoSurveys Geochemical Baseline Database; Geochemical Atlas of Europe. Part 1: Background Information, Methodology and Maps. Espoo: Geological Survey of Finland.) (2005).
37. Lučić M, Mikac N, Bačić N, Vdović N. Appraisal of geochemical composition and hydrodynamic sorting of the river suspended material: Application of time-integrated suspended sediment sampler in a medium-sized river (the Sava River catchment). J Hydrol, 125768 (2020).
38. Dhivert E, Grosbois C, Coynel A, Lefèvre I, Desmet M. Influences of major flood sediment inputs on sedimentary and geochemical signals archived in a reservoir core (Upper Loire Basin, France). CATENA 126, 75-85 (2015).
39. Ollivier P, Radakovitch O, Hamelin B. Major and trace element partition and fluxes in the Rhône River. Chem Geol 285, 15-31 (2011).
40. Davide V, Pardos M, Diserens J, Ugazio G, Thomas R, Dominik J. Characterisation of bed sediments and suspension of the river Po (Italy) during normal and high flow conditions. Water Res 37, 2847-2864 (2003).
41. Zwolsman JJG, van Eck GTM. Geochemistry of major elements and trace metals in suspended matter of the Scheldt estuary, southwest Netherlands. Mar Chem 66, 91-111 (1999).
42. von Eynatten H, Tolosana-Delgado R, Karius V. Sediment generation in modern glacial settings: Grain-size and source-rock control on sediment composition. Sediment Geol 280, 80-92 (2012).
43. Horowitz AJ, Stephens VC. The effects of land use on fluvial sediment chemistry for the conterminous U.S. — Results from the first cycle of the NAWQA Program: Trace and major elements, phosphorus, carbon, and sulfur. Sci Total Environ 400, 290-314 (2008).
44. Takesue RK, Storlazzi CD. Stream sediment geochemistry of four small drainages on the north shore of Kauai west of Hanalei. In: US Geological Survey) (2019).
45. Malkowski MA, Sharman GR, Johnstone SA, Grove MJ, Kimbrough DL, Graham SA. Dilution and propagation of provenance trends in sand and mud: Geochemistry and detrital zircon geochronology of modern sediment from central California (U.S.A.). Am J Sci 319, 846-902 (2019).
46. Granitto M, Schmidt JM, Shew NB, Gamble BM, Labay KA. Alaska Geochemical Database, Version 2.0 (AGDB2)--including “best value” data compilations for rock, sediment, soil, mineral, and concentrate sample media. In: Data Series) (2013).
47. Canfield DE. The geochemistry of river particulates from the continental USA: Major elements. Geochim Cosmochim Acta 61, 3349-3365 (1997).
48. Gaillardet J, Millot R, Dupré B. Chemical denudation rates of the western Canadian orogenic belt: the Stikine terrane. Chem Geol 201, 257-279 (2003).

49. Campodonico VA, García MG, Pasquini AI. The geochemical signature of suspended sediments in the Parana River basin: Implications for provenance, weathering and sedimentary recycling. CATENA 143, 201-214 (2016).
50. Bouchez J, Gaillardet J, France-Lanord C, Maurice L, Dutra-Maia P. Grain size control of river suspended sediment geochemistry: Clues from Amazon River depth profiles. Geochem Geophys Geosyst 12, 1-24 (2011).
51. Rousseau TCC, Roddaz M, Moquet J-S, Handt Delgado H, Calves G, Bayon G. Controls on the geochemistry of suspended sediments from large tropical South American rivers (Amazon, Orinoco and Maroni). Chem Geol 522, 38-54 (2019).
52. Gaiero DM, Brunet F, Probst J-L, Depetris PJ. A uniform isotopic and chemical signature of dust exported from Patagonia: Rock sources and occurrence in southern environments. Chem Geol 238, 107-120 (2007).
53. Hasholt B, Hagedorn B. Hydrology and Geochemistry of River-borne Material in a High Arctic Drainage System, Zackenberg, Northeast Greenland. Arctic, Antarctic, and Alpine Research 32, 84-94 (2000).
54. Thorpe MT, Hurowitz JA, Dehouck E. Sediment geochemistry and mineralogy from a glacial terrain river system in southwest Iceland. Geochim Cosmochim Acta 263, 140-166 (2019).
55. Wimpenny J, James RH, Burton KW, Gannoun A, Mokadem F, Gíslason SR. Glacial effects on weathering processes: New insights from the elemental and lithium isotopic composition of West Greenland rivers. Earth Planet Sci Lett 290, 427-437 (2010).
56. Huang J, et al. Geochemical records of Taiwan-sourced sediments in the South China Sea linked to Holocene climate changes. Palaeogeogr Palaeoclimatol Palaeoecol 441, 871-881 (2016).
57. Liu S, et al. Records of the East Asian winter monsoon from the mud area on the inner shelf of the East China Sea since the mid-Holocene. Chin Sci Bull 55, 2306-2314 (2010).
58. Wang G, Wang Y, Wei Z, He W, Zhang T, Ma X. Geochemical records of Qionghai Lake sediments in southwestern China linked to late Quaternary climate changes. Palaeogeogr Palaeoclimatol Palaeoecol 560, 109902 (2020).
59. Jiwarungrueangkul T, Liu Z, Stattegger K, Sang PN. Reconstructing Chemical Weathering Intensity in the Mekong River Basin Since the Last Glacial Maximum. Paleoceanography and Paleoclimatology 34, 1710-1725 (2019).
60. Wei G, Li X-H, Liu Y, Shao L, Liang X. Geochemical record of chemical weathering and monsoon climate change since the early Miocene in the South China Sea. Paleoceanography 21, (2006).
61. Zhou P, Ireland T, Murray RW, Clift PD. Marine sedimentary records of chemical weathering evolution in the western Himalaya since 17 Ma. Geosphere 17, 824-853 (2021).
62. Wieczorek R, Fantle MS, Kump LR, Ravizza G. Geochemical evidence for volcanic activity prior to and enhanced terrestrial weathering during the Paleocene Eocene Thermal Maximum. Geochim Cosmochim Acta 119, 391-410 (2013).

63. Hessler AM, Zhang J, Covault J, Ambrose W. Continental weathering coupled to Paleogene climate changes in North America. Geology 45, 911-914 (2017).
64. Schoepfer SD, et al. Termination of a continent-margin upwelling system at the Permian–Triassic boundary (Opal Creek, Alberta, Canada). Global Planetary Change 105, 21-35 (2013).
65. Farrell ÚC, et al. The Sedimentary Geochemistry and Paleoenvironments Project. Geobiology 19, 545– 556 (2021).
66. Zhu C, et al. Spore-pollen-climate factor transfer function and paleoenvironment reconstruction in Dajiuhu, Shennongjia, Central China. Chin Sci Bull 53, 42-49 (2008).
67. Cleator SF, Harrison SP, Nichols NK, Prentice IC, Roulstone I. A new multivariable benchmark for Last Glacial Maximum climate simulations. Clim Past 16, 699-712 (2020).
68. Burls NJ, et al. Simulating Miocene Warmth: Insights From an Opportunistic Multi-Model Ensemble (MioMIP1). Paleoceanography and Paleoclimatology 36, e2020PA004054 (2021).
69. Weijers JWH, Schouten S, Sluijs A, Brinkhuis H, Sinninghe Damsté JS. Warm arctic continents during the Palaeocene–Eocene thermal maximum. Earth Planet Sci Lett 261, 230-238 (2007).
70. Wing SL, Harrington GJ, Smith FA, Bloch JI, Boyer DM, Freeman KH. Transient Floral Change and Rapid Global Warming at the Paleocene-Eocene Boundary. Science 310, 993-996 (2005).
71. Joachimski MM, et al. Climate warming in the latest Permian and the Permian–Triassic mass extinction. Geology 40, 195-198 (2012).

Part II Spatial distribution of WIP and its controls

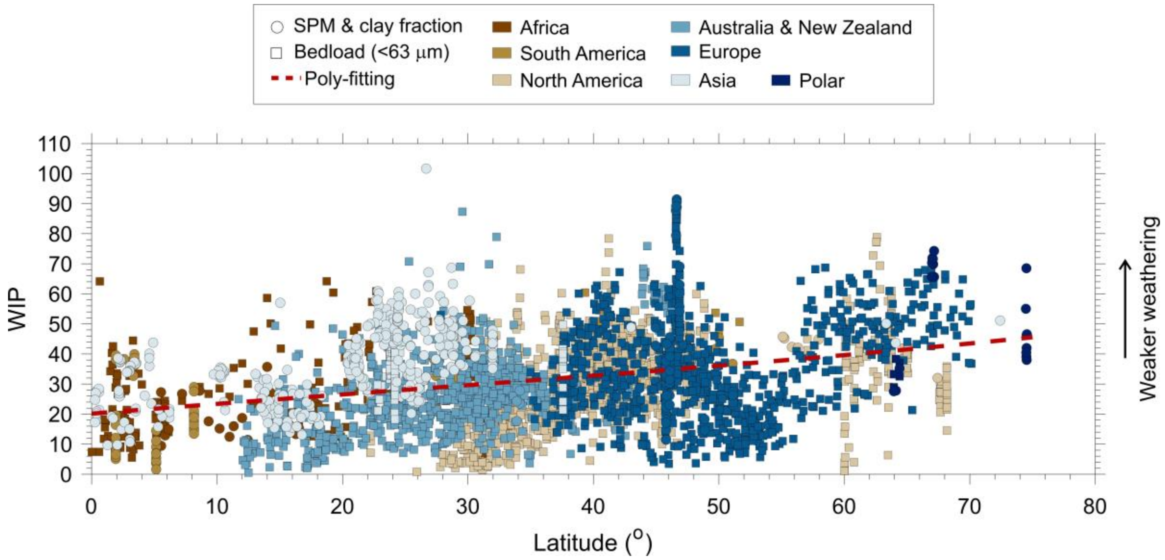

Supplementary Fig. 1 Latitudinal distribution of WIP in fine-grained sediments. Sample types include surface suspended particulate matter (SPM), clay fraction of sediments and bedload sieved to <63 μm. The latitude of sampling locations shown on the X-axis equally refers to the southern and northern hemispheres, respectively. The polynomial fitting between latitude and weathering index (red dashed line) is applied. In general, WIP increases with latitude. "Polar" symbols here include samples from Greenland and Iceland. Data source is the same as that in Fig. 2 of the main text.

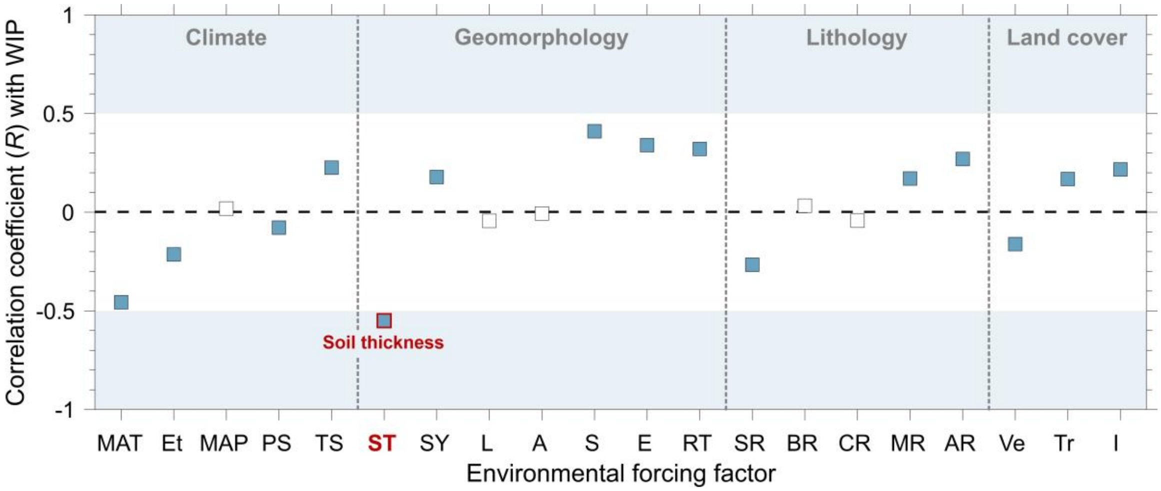

Supplementary Fig. 2 Correlation coefficients (*R*) between each environmental forcing factor (n=20) and WIP. Correlations with p value of >0.001 are shown as open symbols. The order of forcing factors in each category is sorted by correlation coefficient with CIA (Fig. 3 in the main text). Negative values mean a negative correlation. Upland hillslope soil thickness is the only factor with $|R|$ of >0.5.

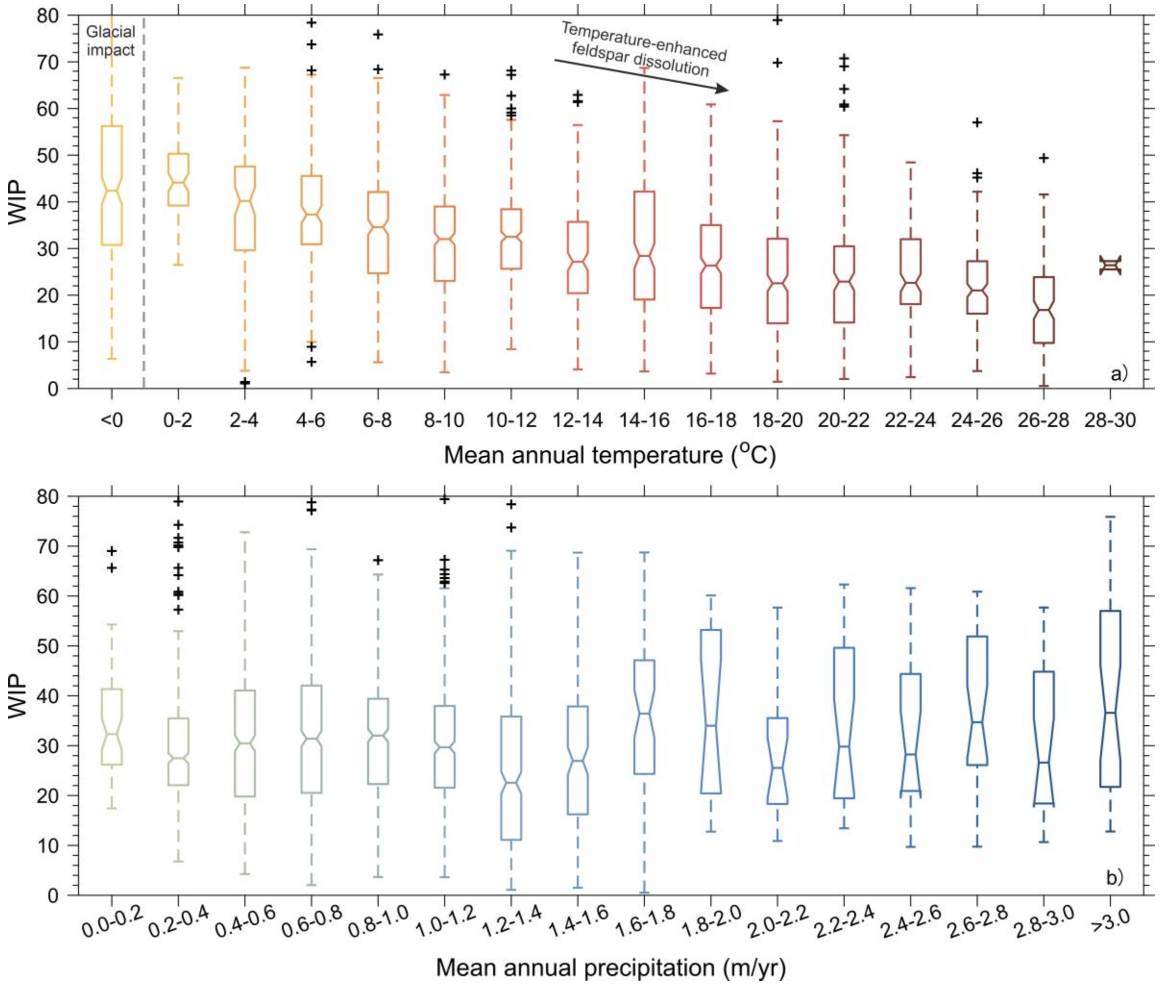

Supplementary Fig. 3 Boxplots of WIP grouped by zones of MAT (a, interval of 2 °C) and MAP (b, interval of 0.2 m/yr). WIP decreases with MAT over a large temperature gradient and shows a more complicated pattern with MAP.

Part III Effect of lithology, mineralogy, and sediment grain-size on weathering index

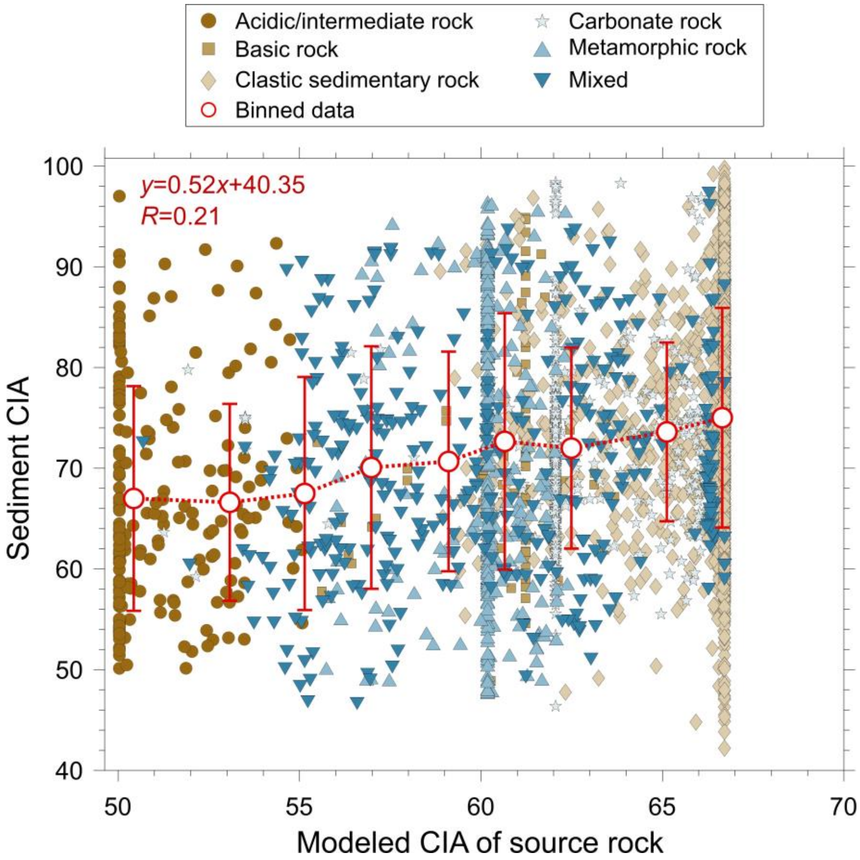

Supplementary Fig. 4 Relationship between modeled CIA of source rock and sediment CIA. The fitting equation is shown in the plot. The red open circles are sediment CIA data binned by modeled CIA of source rock (interval of 2 units) and error bars indicate one standard deviation. Modeled CIA of source rock is calculated using areal percentage of each lithology in a sampling basin and the representative major elemental concentrations of each lithology (Supplementary Data 2). The symbol assignment is based on the lithology of each sampling basin: an areal percentage of >60% for a single lithology is plotted using the corresponding lithological symbol; if all lithologies are <60% in area, it is characterized as a "mixed" symbol. The calculation of CIA only accounts for the silicate fraction (Methods) and thus CIA of carbonate rocks is controlled by the minor contribution (<40%) of other lithologies. Binned sediment CIA data does not monotonically increase with source-rock CIA, and its variability at any interval of source-rock CIA is quite large (standard deviation of ~11 units) due to discrepancy in chemical weathering processes. Hence, although CIA can vary between rock types, the control of source rock on CIA in fine-grained sediments is only subordinate given the rather weak positive correlation (R=0.21).

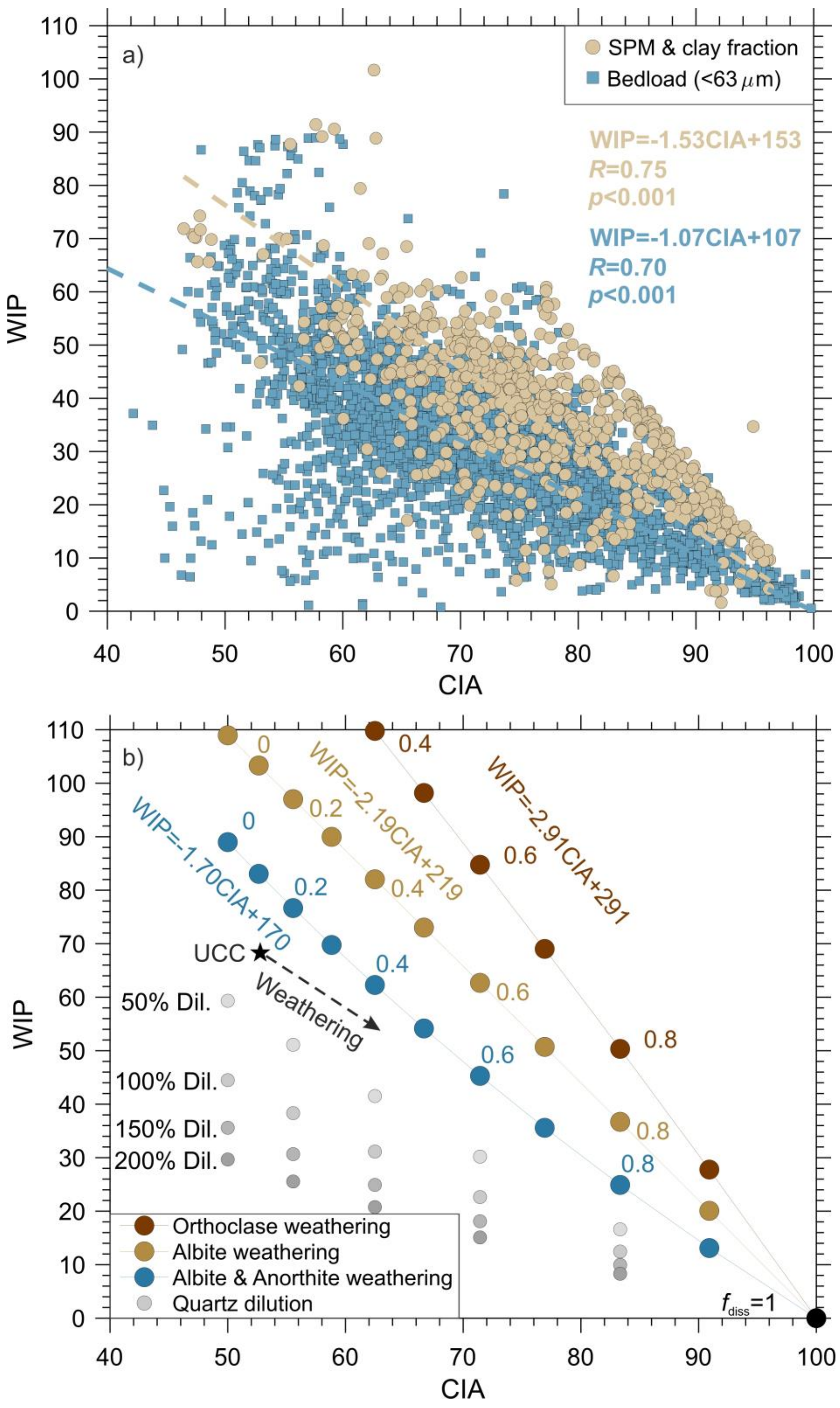

Supplementary Fig. 5 CIA vs. WIP. a) The CIA-WIP diagram of fine-grained sediment samples and b) a conceptual diagram of the CIA-WIP correlation for weathering of diverse mineral sources including different types of feldspar and quartz addition. In b) numbers (0-1) next to each fitting line indicate the dissolution percentage of each mineral

(f_{diss}). The "Albite & Anorthite weathering" is characterized by an original molar ratio of albite to anorthite as 2[72]. Numbers next to grey circles (quartz dilution) indicate the amount of quartz added to the mineral mixture weathered from albite and anorthite. For example, "100% Dil." means that the mass of quartz added is the same as that of the weathered mixture. Examples of quartz dilution effect are shown for f_{diss} of 0%, 20%, 40%, 60% and 80%. UCC means upper continental crust[73]. Note that different parent materials shown in b) follow different weathering trends characterized by slopes of CIA-WIP relation[74]. In our compiled dataset (a), the fitting slope of SPM and clay fraction is close to that of plagioclase (albite and anorthite) weathering (b), consistent with the notion that the mineralogical composition of upper continent crust is dominated by plagioclase (~40%)[75]. The lower fitting slope of bedload (<63 μm) may suggest addition of different amounts of quartz and/or weathering of other minerals. However, quartz dilution does not affect CIA values and reduce WIP only (b).

Part IV Relationship between climatic factors and weathering index

Supplementary Table 1 Effect of precipitation on weathering indices. Correlation coefficient (*R*) and *p* value between mean annual precipitation (MAP) and weathering indices (CIA-WIP) for each given zone of MAT (interval of 5 °C) are calculated. $|R| > 0.5$ is only found for WIP when MAT<0 ºC.

MAT zone	CIA		WIP		*N*
	R	*p*	*R*	*p*	
<0	-0.28	<0.001	0.54	<0.001	196
0-5	-0.06	0.363	0.02	0.732	236
6-10	-0.01	0.690	0.32	<0.001	730
11-15	0.26	<0.001	-0.04	0.357	575
16-20	0.39	<0.001	-0.02	0.625	654
21-25	0.31	<0.001	0.05	0.325	395
26-30	0.24	<0.001	0.20	0.005	203

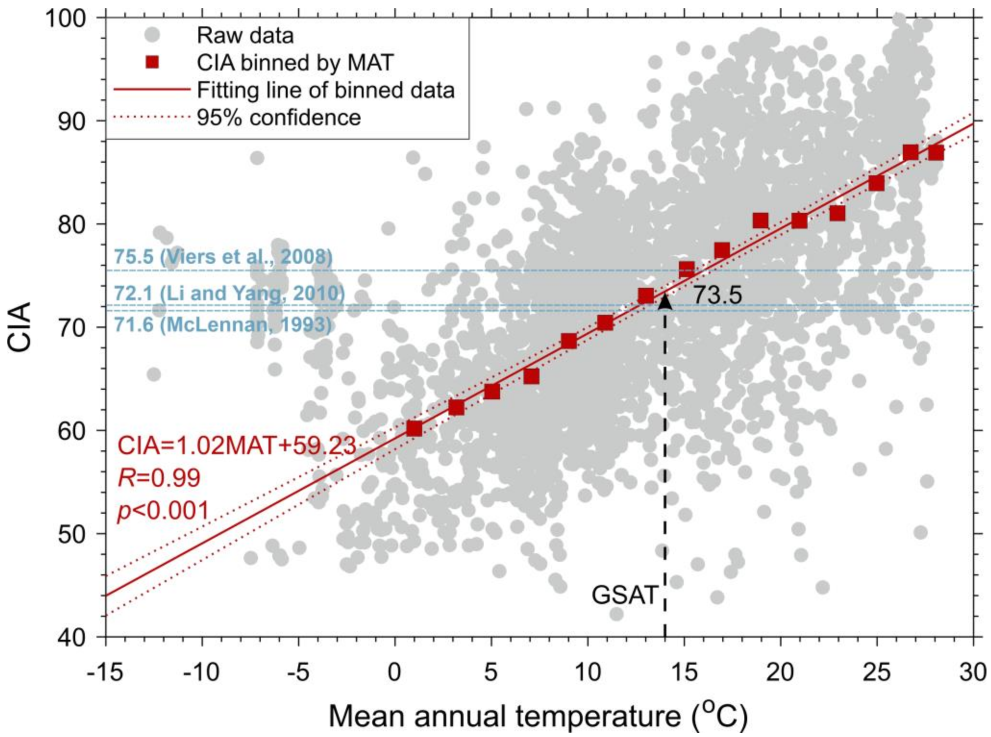

Supplementary Fig. 6 Empirical relationship between MAT and CIA in fine-grained sediment. CIA data are binned by MAT (an interval of 2 ºC, MAT >0 ºC; shown as red squares) to derive the fitting equation. The corresponding CIA at global mean surface air temperature (GSAT, 14 °C)[76] is 73.5, falling within the range of previous estimates of global-average CIA (71.6-75.5)[72, 77, 78] using mainly suspended sediments in large rivers.

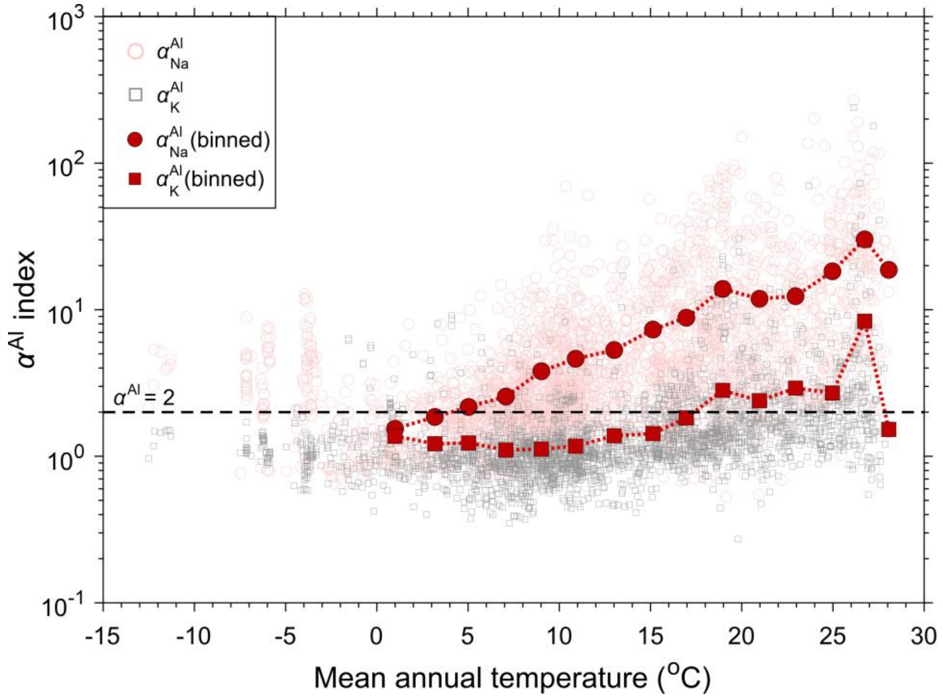

Supplementary Fig. 7 Relationship between α^{Al} index (α^{Al}_{Na} and α^{Al}_{K}) and MAT in fine-grained sediment. Note that y-axis is on log scale. α^{Al} indicates the mobility of one element during weathering and a higher value suggests a stronger element depletion. The equations for calculating such index are: α^{Al}_{Na} =$(Al/Na)_{sample}/(Al/Na)_{UCC}$ and α^{Al}_{K}=$(Al/K)_{sample}/(Al/K)_{UCC}$[23]. Raw data are open symbols and average index data binned by MAT (an interval of 2 ^oC, MAT >0 ^oC) are filled symbols. In general, binned α^{Al}_{Na} increases with MAT across a large temperature gradient and becomes higher than e.g. 2 (50% depletion compared to [Na] in UCC) when MAT>~5 ^oC, while binned α^{Al}_{K} scatters around 1 at MAT of 0-15 ^oC and exceeds 2 when MAT>~20 ^oC. It suggests that weathering of plagioclase (e.g. albite) starts from a much lower temperature while significant release of K (in orthoclase) only occurs at MAT > ~20 ^oC.

Supplementary Table 2 A sensitivity test on MAT-CIA relationship (in the format of "CIA=*a**MAT+*b*"). Effects of grain-size, mineralogical composition (indicated by slope of CIA-WIP relation) and dominant lithology are examined.

	a[1]	***b***[1]	***R***[1]	***N***[1]
Grouped by sediment grain-size				
SPM or clay fraction	0.92 ± 0.11	64.48 ± 2.23	0.72	265
Bedload (<63 μm)	1.02 ± 0.05	59.06 ± 0.77	0.61	2528
Grouped by slope of CIA-WIP relation				
0-0.5[2,3]	0.41 ± 0.36	60.55 ± 4.26	0.19	132
0.5-1	0.92 ± 0.08	58.77 ± 1.22	0.62	816
1-1.5	0.97 ± 0.07	59.71 ± 0.97	0.61	1281
1.5-2	0.87 ± 0.14	65.79 ± 2.63	0.52	373
>2[3]	0.61 ± 0.14	74.15 ± 3.07	0.52	191
Grouped by dominant lithology				
Igneous	0.95 ± 0.16	59.32 ± 1.96	0.58	258
Clastic sedimentary & metamorphic	1.05 ± 0.06	58.93 ± 0.97	0.64	1845
Carbonate[4]	1.23 ± 0.19	57.61 ± 2.34	0.59	311
Mixed lithology[5]	1.00 ± 0.12	59.20 ± 1.70	0.66	354
Summary				
All data[6]	1.05 ± 0.05	58.98 ± 0.72	0.64	2793

Note: 1. *a* and *b* are coefficients of the best-fit line, and the standard deviation indicates the 95% confidence bounds. *R* and *N* are correlation coefficient and number of samples, respectively.

2. *p* values of all data groups are < 0.01 except the group with a slope of CIA-WIP relation of 0-0.5.

3. The lower *a* of both groups may not be representative over continental scale, because their small *N* (<200) can result in a larger uncertainty in fitting and/or a potential bias toward specific environmental conditions.

4. Such CIA values are dominated by minor outcrops of non-carbonate rock, and the link of sediment CIA with basin-averaged MAT may be less direct. Nevertheless, its relation slope still agrees with those of other lithological groups within uncertainty.

5. If each lithology in one catchment is <60% in area, it is characterized as mixed lithology.

6. Dataset used for this sensitivity test only include samples with a MAT of >0 ^oC.

References

72. McLennan SM. Weathering and Global Denudation. The Journal of Geology 101, 295-303 (1993).
73. Rudnick RL, Gao S. 3.01 - Composition of the Continental Crust A2 - Holland, Heinrich D. In: Treatise on Geochemistry (ed Turekian KK). Pergamon (2003).
74. Ohta T, Arai H. Statistical empirical index of chemical weathering in igneous rocks: A new tool for evaluating the degree of weathering. Chem Geol 240, 280-297 (2007).
75. Nesbitt HW, Young GM. Prediction of some weathering trends of plutonic and volcanic rocks based on thermodynamic and kinetic considerations. Geochim Cosmochim Acta 48, 1523-1534 (1984).
76. Hansen J, Ruedy R, Sato M, Lo K. Global Surface Temperature Change. Rev Geophys 48, (2010).
77. Viers J, Dupré B, Gaillardet J. Chemical composition of suspended sediments in World Rivers: New insights from a new database. Sci Total Environ 407, 853-868 (2009).
78. Li C, Yang S. Is chemical index of alteration (CIA) a reliable proxy for chemical weathering in global drainage basins? Am J Sci 310, 111-127 (2010).
