# Supplementary material for: A global temperature control of silicate weathering intensity
Source: Nat Commun. 2022 Apr 4;13:1781. doi: 10.1038/s41467-022-29415-0 (PMC8980099; doi:10.1038/s41467-022-29415-0)
Supplement: Supplementary file 2 — Description of Additional Supplementary Files [file 41467_2022_29415_MOESM2_ESM.pdf]

Description of Additional Supplementary Files

File Name: Supplementary Data 1

Description: Dataset of major elements in the river sediments investigated in this study (*n*=3828)

File Name: Supplementary Data 2

Description: Dataset of environmental parameters extracted for selected sediment samples (*n*=2989)

File Name: Supplementary Data 3

Description: Information on selected sediment archives for reconstructing paleo-temperature using MAT-CIA equation. The corresponding biomarker/pollen-derived paleo-temperature from literature is also provided.

Note that Supplementary Data 1-3 above are provided in the spreadsheets of "Supplementary Dataset.xlsx".
